# Frailty as a Predictor of Cognitive Disorders: A Systematic Review and Meta-Analysis

**DOI:** 10.3389/fmed.2019.00026

**Published:** 2019-02-19

**Authors:** Marcus Kiiti Borges, Marco Canevelli, Matteo Cesari, Ivan Aprahamian

**Affiliations:** ^1^Department and Institute of Psychiatry, Faculty of Medicine, University of São Paulo, São Paulo, Brazil; ^2^Department of Human Neuroscience, Sapienza University, Rome, Italy; ^3^Fondazione IRCCS Ca' Granda Ospedadale Maggiore Policlinico, University of Milan, Milan, Italy; ^4^Geriatrics and Psychiatry Division, Department of Internal Medicine, Faculty of Medicine of Jundiaí, Jundiaí, Brazil

**Keywords:** mild cognitive impairment, cognitive decline, dementia, cognitive disorders, comorbidity, elderly, meta-analysis

## Abstract

**Background/Aim:** Current evidence in the literature supports associations between frailty, cognitive impairment, and dementia. The study aim was to describe the risk of cognitive disorders associated with physical frailty in older adults from community-based studies.

**Methods:** We performed a systematic review and meta-analysis, using MEDLINE, PsycINFO, Scopus, and Web of Science as databases for the search. Cohort and longitudinal studies were included in qualitative analysis and quantitative synthesis. For inclusion, studies had to assess dementia and cognitive impairment as a primary or secondary outcome, and describe the prevalence of frailty among participants at baseline and follow-up.

**Results:** Of the 2,210 studies retrieved by the systematic review, 6 relevant studies were included in a meta-analysis. Baseline frailty was significantly associated with an increased risk of geriatric cognitive disorders (pooled OR = 1.80, 95% CI = 1.11–2.92; *p* = 0.02). Heterogeneity across the studies was significant (*I*^2^ = 79%).

**Conclusions:** The analyses confirmed that frail older adults were at higher risk of incident cognitive disorders than non-frail elders. Frailty status seems to be most associated with the risk of incident dementia. Frailty may represent a risk factor for dementia and could constitute a novel modifiable target in early cognitive impairment.

## Introduction

From its definition, frailty can be understood as a state of higher vulnerability to stressors attributed to a lower homeostatic reserve due to an age-related multisystem physiological change (1). Frailty refers to a potentially reversible pathological aging process that occurs at an intermediate stage between aging-related diseases (senility) and relevant adverse outcomes such as disability and death (2). It is a common geriatric condition with a mean prevalence of 10% (3). Gill et al. conducted a study investigating risk factors associated with disability in the last year of life and reported that frailty was the condition most frequently leading to death (4).

Several types of operational definitions have emerged contributing to the diagnosis of frailty, ranging from physical or phenotype criteria [e.g., Fried's phenotype criteria (1)] to multidimensional models [e.g., Frailty Index (5)]. A third frailty model warranting special attention is the biopsychosocial model (another multidimensional model) which combines physical and psychosocial domains (6). This construct is oriented toward the social sciences and emphasizes the importance of an integral conceptual definition of frailty (7). In general, independently of the validated criteria used, the diagnosis of frailty is associated with adverse health outcomes (falls, disability, hospitalization, institutionalization, or death) (2).

Current evidence in the literature from cross-sectional and longitudinal studies has shown relationships between frailty and cognitive disorders (including mild cognitive impairment and dementia) (8–10). Frailty may increase the future risk of mild cognitive impairment (MCI) and all-cause dementia in cognitively unimpaired populations, as well as accelerate cognitive decline of these individuals (11). Furthermore, components of frailty appeared to be related to pathological findings of Alzheimer's disease (AD) and vascular dementia, supporting the notion of a possible common biological pathway between frailty and cognitive disorders (12, 13). Despite this evidence, there is debate over the magnitude of the association between frailty and cognitive impairment. Some longitudinal studies show that frailty is associated with dementia, especially vascular dementia (14–16). Frailty was identified retrospectively, or using non-validated criteria, in many other studies (10, 13, 17, 18). Additionally, in previous systematic reviews and meta-analyses, more recently published studies were not included and the number of incident cognitive impairment cases among frail participants was not clearly reported (19–21).

Interest in this field of research has been growing rapidly in the past 5 years (22, 23). It is thus essential to define the relevant aspects that are useful for the definition of the construct of cognitive frailty for use in both clinical practice and research (22, 23). Therefore, the understanding of the relationship between frailty and geriatric cognitive disorders could contribute to new interventions for the prevention and management of both conditions. Finally, the main objective of this systematic review and meta-analysis was to describe the risk of development of cognitive disorders in previously cognitively unimpaired community-dwelling older adults or those with MCI associated with frailty at baseline from longitudinal and cohort studies.

## Methods

### Data Source and Search Strategy

A systematic literature search of PubMed (MEDLINE), SCOPUS, PsycINFO, and Web of Science from 1st March 2001 through January 2018 was conducted according to the Standards for Systematic Reviews (24) and the Preferred Reporting Items for Systematic Review and Meta-Analyses (PRISMA) guidelines (25). The publication period was decided based on the most widely used definition of frailty, Fried's phenotype criteria (1), published on 1st March, 2001. In addition to the date limit, the following filters were used: English language, humans, aged 65 years or older. The inclusion criteria were: (i) older adults without dementia at baseline; (ii) community-dwelling population; (iii) cohort or longitudinal studies; (iv) frailty defined according to common, validated and recognized criteria, and evaluated prospectively; (v) incidence of geriatric cognitive disorders at the end of a follow-up of at least 2 years; and finally; (vi) if dementia was the main outcome, it had to be diagnosed based on well-known established criteria such as the Diagnostic and Statistical Manual of Mental Disorders (DSM) (26) or National Institute of Neurological and Communicative Disorders and Stroke, Alzheimer's Disease and Related Disorders Association (NINCDS-ADRDA) (27) or National Institute of Neurological Disorders and Stroke, Association Internationale pour la Recherche et l'Enseignement en Neurosciences (NINDS-AIREN) (28).

The search terms used included the following: [(“cognition”[MeSH] OR “cognition”) OR (“cognitive dysfunction”[MeSH] OR (“cognitive” AND “dysfunction”) OR “cognitive dysfunction” OR (“mild” AND “cognitive” AND “impairment” OR “mild cognitive impairment”) OR (“dementia”[MeSH] OR “dementia”)] AND [(“frailty”[MeSH] OR “frailty”) OR (“frail elderly”[MeSH] OR (“frail” AND “elderly”) OR “frail elderly”)]. The bibliographies of relevant reviews and meta-analyses involving frailty and cognitive impairment were also manually searched and additional references obtained from outside experts.

### Study Selection

Two independent authors reviewed each study abstract according to the inclusion criteria, and the full text of all studies retrieved by the literature search for eligibility. Cohort and longitudinal studies that assessed dementia and cognitive impairment as a primary or secondary outcome and described the prevalence of frailty among participants at baseline were included in the quantitative synthesis. Only studies conducted among community-dwelling older adults were included. Studies that were reviews, editorials or letters, clinical, and cross-sectional studies were excluded. Any disagreement over studies selected by any of the authors was resolved by consensus of the authors involved.

### Data Extraction and Quality Assessment

Two authors extracted the data according to a predefined format for presentation: author, year; population, exposures, comparators, outcomes, and study design. The authors abstracted study design information, population characteristics at baseline, exposure details, disease prevalence at baseline, and incidence at the end of follow-up, and risk estimates such as OR (Odds ratio) or HR (Hazard ratio) with 95% confidence intervals (95%CI) from all included studies into a standardized table. Two authors assessed the quality and risk of bias for each study included in the qualitative analysis. The Newcastle-Ottawa Quality Assessment Scale (29) was used for this evaluation of quality, where each study was assessed for good standards on four items of selection, one item of comparability, and three items of outcome, yielding a total of 8 stars (points) (comparability can be scored with up to two stars).

### Data Synthesis and Analysis

All evidence drawn from the studies was described qualitatively and summarized in Table 1. We also analyzed the results from the studies using quantitative estimates of effects by the Mantel-Haenszel method. Thus, a random-effects meta-analysis was conducted to estimate the odds ratio of cognitive decline between frail and non-frail participants using the RevMan software, version 5.3 (Review Manager (RevMan) [Computer program]. Version 5.3. Copenhagen: The Nordic Cochrane Center, The Cochrane Collaboration, 2014). Confidence interval was set at 95%, and the level of significance was set at <5%.

**Table 1:** Summary of studies included in the qualitative analysis.

**Author, year/Country population**	**Exposures**	**Comparator (control)**	**Outcomes**	**Study design/follow-up**	**NOS Grade**
Avila-Funes 2012 (14) France 5,480 older adults (aged 65–95) Mean age = 74 years	PF: Fried phenotype (mCHS). Cognitive assessment: MMSE, IST; neuropsychological testing. Diagnosis of dementia: according to DSM-IV and NINDS-AIREN criteria.	Compared frail vs. non frail (HRs were adjusted for dementia risk factors cardiovascular risk factors)	Incidence of dementia. Frailty was associated with greater risk of all dementia (HR = 1.24; 95% CI: 0.94–2.01) and VaD (HR = 2.73; 95% CI: 1.05–7.13); not related to AD dementia.	Population-based longitudinal study 7 years	8/9
Feng 2017 (30) Singapore 1,575 older adults from the SLAS-1 sample of 2,611 participants Mean age = 66 years	PF: Fried phenotype (mCHS). Cognitive assessment: MMSE, CDR. Depressive symptoms: GDS-5. Diagnosis of NCD according DSM-5 criteria.	Compared robust and CN subjects to prefrail or frail older adults with cognitive impairment. (controlled for age, gender, education, APOE-e4, MMSE, CHF, AF, diabetes, smoking, alcohol use, depressive symptoms)	Incidence of Cognitive Impairment and NCD. PF was associated with increased incident cognitive impairment (OR = 4.43; 95% CI: 0.77–25.7) and greater risk of incident NCD (OR = 6.37; 95% CI : 1.74–23.28).	Population-based longitudinal study 3 years	8/9
Gray 2013 (15) USA 2,619 participants (aged 65 or older) Mean age = 76.8 years	PF: Fried phenotype (CHS). Cognitive assessment: CASI, neuropsychological testing. Diagnosis of dementia: DSM-IV and NINCDS-ADRDA criteria.	Compared frail *vs*. robust participants. (HRs were adjusted for age, sex, education, depressive symptoms, antidepressant use, BMI, self-rated health, hypertension, diabetes, cardiovascular disease, smoking and cognitive status)	Incidence of all-cause dementia, possible or probable AD, non-AD dementia. Frailty was associated with higher risk of non-AD dementia (aHR = 2.57; 95% CI: 1.08–6.11) and all-cause dementia (aHR = 1.20; 95% CI: 0.85–1.69), not related to AD dementia.	Cohort 6.5 years	8/9
Montero-Odasso 2016 (32) Canada 252 older adults from the “Gait and Brain Study” Mean age = 76.7 years	PF: Fried phenotype (CHS). Cognitive assessment: MoCA. Physical examination and clinical evaluation for comorbidities, medications, physical activity level, ADL and IADL questionnaires. Diagnosis of dementia: DSM-IV and CDR.	Compared PF alone *vs*. PF combined with cognitive impairment and each of five factors of PF combined with baseline cognitive status. (HRs were adjusted for age, sex, education and comorbidities)	Risk of cognitive decline and incident dementia. The combination of slow gait and cognitive impairment showed the highest risk for progression to dementia (HR = 35.9; 95% CI: 4.0–319.2).	Population-based longitudinal study 5 years	7/9
Solfrizzi, 2013 (16) Italy 2,581 older adults from the ILSA sample of 5,632 (aged 65–84) Mean age = 73.1 years	PF: Fried phenotype (mCHS). Cognitive assessment: MMSE. Clinical evaluation, Physical activity questionnaire, CCI, depressive symptoms (GDS-30), ADL and IADL scale. Diagnosis of dementia: DSM-IIIR, NINCDS-ADRDA, ICD-10.	Compared frailty and risk of incident dementia. (HRs were adjusted for age, sex, education, smoking, IADL, MMSE, CCI, and albumin levels)	Incidence of dementia, AD, and VaD dementia. Frailty was associated with an increased risk of overall dementia (aHR = 1.85; 95% CI: 1.01–3.40) and VaD (aHR = 2.68; 95% CI:1.16–7.17)	Population-based longitudinal study 3.5 years	8/9
Solfrizzi 2017 (31) Italy 2,150 older adults from the ILSA sample of 5,632 (aged 65-84) Mean age = 73.2 years	PF: Fried phenotype (mCHS). Cognitive assessment: MMSE. Clinical examination for CAD, CHF, T2DM, hypertension, stroke; smoking, IADL scale, depressive symptoms (GDS-30), CCI. Diagnosis of dementia: DSM-IIIR, NINCDS-ADRDA, ICD-10.	Compared the risk of incident dementia over 3.5 years *vs*. 7 years. (HRs were adjusted for age, sex, education, smoking, IADL, MMSE, GDS, CCI, and albumin levels)	Incidence of dementia, its subtypes, and mortality. Cognitive Frailty was predictor of overall dementia, particularly VaD (HR = 2.30; 95% CI: 1.02–5.18) *vs*. (HR = 2.12; 95% CI: 1.12–4.03).	Population-based longitudinal study 3.5 and 7 years	9/9

*NOS. Newcastle-Ottawa scale; PF. physical frailty; mCHS. modified Cardiovascular Health Study; MMSE. Mini-Mental State Examination; IST. Isaac Set Test; DSM-IV. Diagnostic and Statistical Manual of Mental Disorders, 4th edition; NINDS-AIREN. National Institute of Neurological Disorders and Stroke - Association Internationale pour la Recherche et l'Enseignement en Neurosciences; VaD. Vascular dementia; AD. Alzheimer's disease; HR. Hazard ratio; SLAS. Singapore Longitudinal Ageing Studies; CDR. Clinical dementia rating; GDS-5. Geriatric Depression Scale (5-item); NCD. Neurocognitive disorders; USA. United States of America; DSM-5. Diagnostic and Statistical Manual of Mental Disorders, 5th edition; CN. cognitively normal; APOE-e4. Apolipoprotein E e4; CHF. congestive heart failure; AF. atrial fibrillation; CHS. Cardiovascular Health Study; CASI. Cognitive Abilities Screening Instrument; BMI. body mass index; MoCA. Montreal Cognitive Assessment; ADL. activities of daily living; IADL. instrumental activities of daily living; ILSA. Italian Longitudinal Study on Aging; GDS-30. Geriatric Depression Scale (30-item); CCI. Charlson comorbidity index; DSM-IIIR. Diagnostic and Statistical Manual of Mental Disorders, 3th edition revised; NINDS-ADRDA. National Institute of Neurological and Communicative Disorders and Stroke - Alzheimer's Disease and Related Disorders Association; ICD-10. International Statistical Classification of Diseases and Related Health Problems, 10th revision; CAD. coronary artery disease; T2DM. type 2 diabetes mellitus*.

## Results

### Selection Process

**Figure 1:**
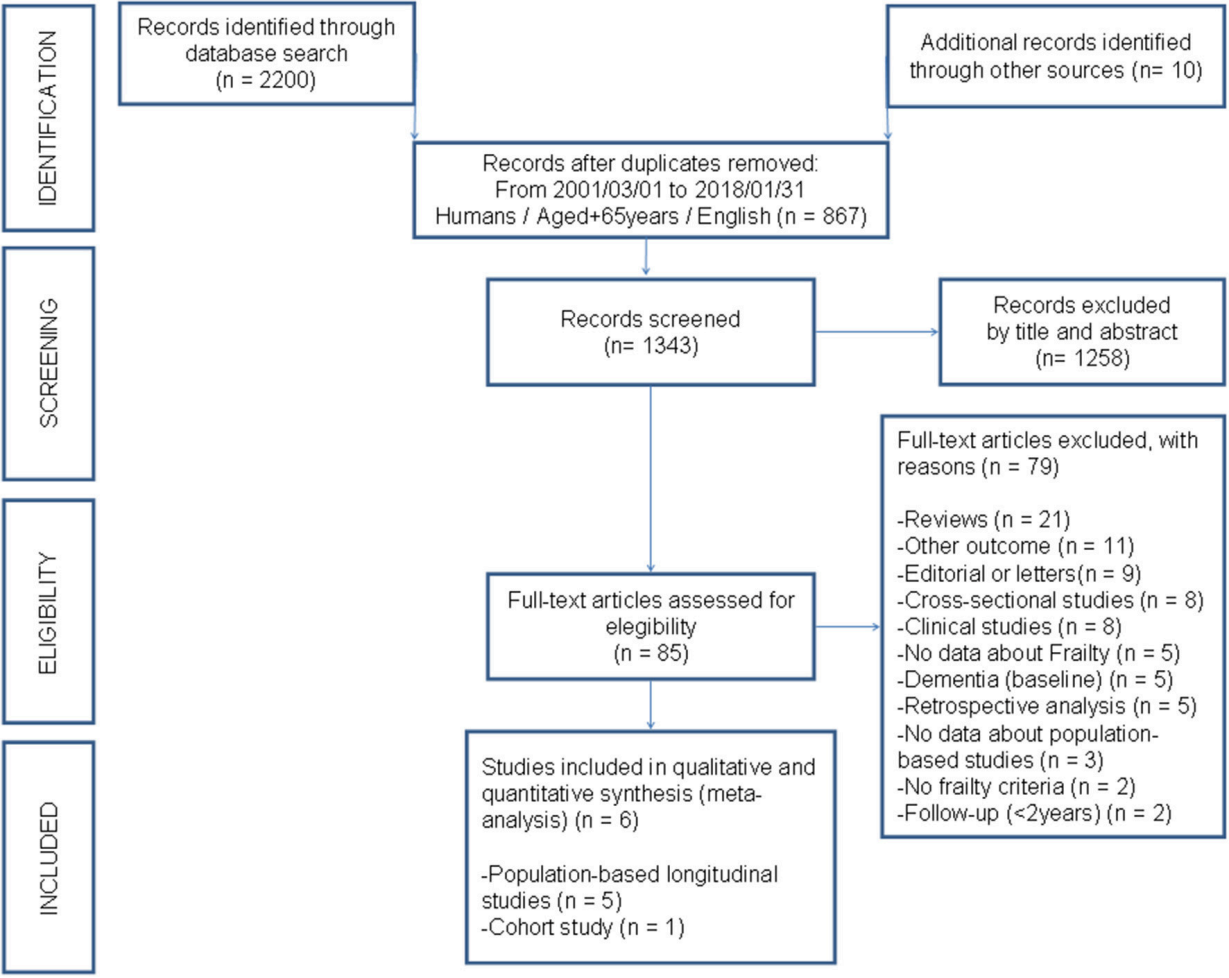
Flowchart of the systematic search.

The systematic search of the literature yielded 2,200 citations. A further 10 studies had not been identified and were added manually. Of the 2,210 records, 867 studies were removed by search filters: publication period (March, 2001 to January, 2018), English language, humans, aged over 65 years. Of the 1,343 records, 1,258 studies considered not relevant were excluded, giving a total of 85 studies for full-text review. Twenty-one reviews, nine editorials or letters; eight clinical studies; and eight cross-sectional studies were subsequently excluded. Five studies were excluded for not categorizing frailty status or showing an association with MCI and dementia. Of the remaining thirty-four studies, twenty-eight were excluded for not fulfilling the criteria for this study. The complete list of excluded studies can be found in the Supplementary File. The remaining six studies were considered to have adequate methodological quality and included in the qualitative and quantitative syntheses (meta-analysis). Figure 1 depicts the flowchart of the study selection process.

### Study and Participant Characteristics

Study and participant characteristics of the cohort or population-based longitudinal studies are summarized in Table 1. The studies were conducted among community-dwellers in North America (*n* = 2); Europe (*n* = 3); and Asia (*n* = 1). Sample size ranged from 1,575 to 5,480 (total of 14,657 participants). Mean age of study participants was 73.3 years. The overall quality of the studies assessed using NOS was high, with a median score of 8 (Table 1).

### Longitudinal Meta-Analysis Findings

**Figure 2:**
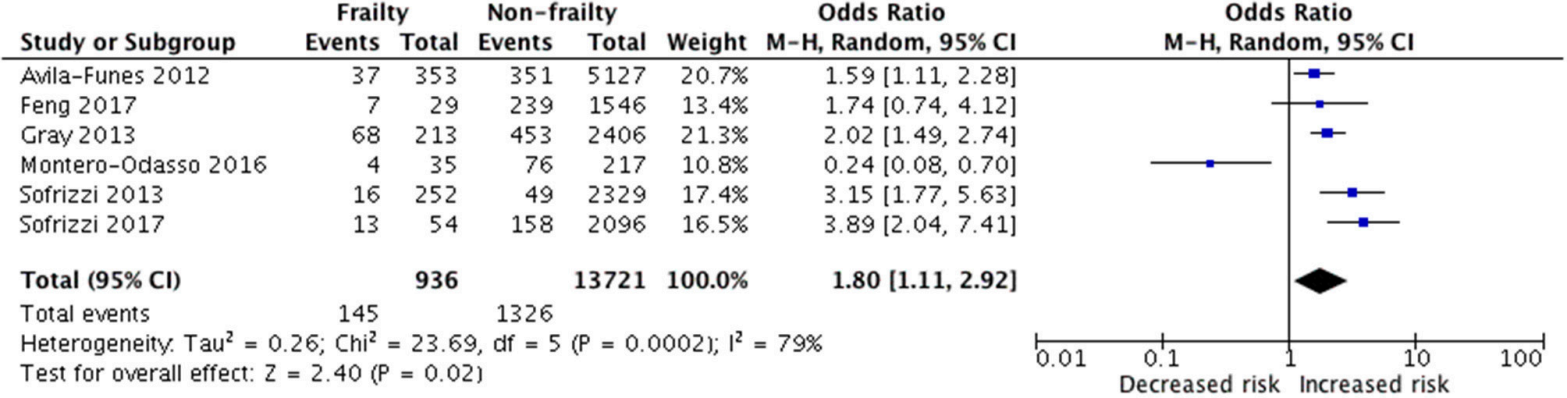
Random-effects meta-analysis of incident cognitive disorder associated with frailty in older adults.

There were 936 frail older adults in the 6 studies (14–16, 30–32) investigating the incidence of cognitive disorders over a mean follow-up of 5.33 years (range 3 to 7 years). These subjects were compared with 13,721 non-frail individuals at baseline (Figure 2).

Results showed that baseline frailty was significantly associated with an increased risk of geriatric cognitive disorders (pooled OR = 1.80, 95% CI = 1.11–2.92 *p* = 0.02; I^2^ = 79%), as shown in Figure 2. Heterogeneity across the studies was significant (I^2^ = 79%).

### Frailty and the Risk of Geriatric Cognitive Disorders

Frail status was most associated with the risk of dementia, particularly non-AD and vascular dementia, even after adjusting for many confounders, as shown in Table 1.

In the Three-City study (14), frailty was a major risk factor for incident dementia and was associated with greater risk of all types of dementia. In the ILSA study (16), physical frailty was associated with a significantly increased risk of overall dementia and vascular dementia over a 3.5-year follow-up, while the risk of AD or other types of dementia did not significantly change in frail individuals compared with robust older adults. Later studies confirm the impact of frailty on incident vascular and overall dementia, but not AD dementia (15, 31). Frail participants did not exhibit a significant risk for incident dementia in the Gait and Brain Study (32).

There were major disparities in definitions of cognitive impairment and assessments of cognitive functioning. Most studies employed different methods (e.g., MMSE, MoCA) and cut-off values for defining cognitive impairment. Several studies evaluated the cognitive performance of participants using screening measures of global cognition. Only two studies adopted a comprehensive neuropsychological test battery (14, 15) and CDR scale (30, 32). Only one study showed that PF was associated with both incident cognitive impairment and greater risk of neurocognitive disorders (NCD) in older adults (30).

## Discussion

In this systematic review and meta-analysis, the relationship between frailty, and cognitive disorders was investigated, summarizing data from longitudinal and cohort studies involving community-dwelling older adults. Our analyses confirmed that frail older adults were at higher risk of incident cognitive disorders, especially vascular dementia, compared with non-frail elders. Previous longitudinal studies have reported that physical frailty may be associated with incident vascular dementia (14, 16). In fact, physical frailty was associated with increased risk of developing vascular dementia in three of the studies included in the present systematic review (14, 16, 31).

Vascular dementia is caused by cardiovascular disease (CVD). It has been suggested that CVD and vascular cognitive impairment (cerebrovascular disease) in the elderly have the same risk factors (33). Frailty has been associated with an increased odds for hypertension and diabetes (34, 35). Atrial fibrillation (AF) is another major risk factor for cerebrovascular disease. A recent systematic review investigating the association between AF and frailty shows that a higher prevalence of frailty was observed among patients with this CVD (36). Veronese et al. conducted a study showing that frailty is an independent risk factor for any-type of CVD in older adults (37). Moreover, studies have shown that obesity and metabolic disorders are associated with cognitive decline and dementia (38–40). Metabolic Syndrome and insulin resistance are associated with increased risk of frailty (41). However, current evidence on Metabolic Syndrome and risk for cognitive decline in the elderly is conflicting (42).

Physical frailty has been associated with late-life cognitive decline, incident AD and mild cognitive impairment, vascular dementia, and with non-AD dementia in older adults according to findings of previous systematic reviews (19–21). Several studies examining frailty and cognitive impairment suggest these outcomes interact and the existence of a possible bidirectional relationship (23). A pooled prevalence of physical frailty of 32% in patients with AD was reported in a previous systematic review (43).

Cognitive impairment has been considered either a syndrome (e.g., MCI, Subjective Cognitive Decline (SCD), NCD, or cognitive frailty when combined with frailty diagnosis) or a preclinical stage of AD (prodromal AD or preclinical AD) (44). Moreover, studies show a higher prevalence of cognitive impairment among frail older people (45). In our review, we found two studies that considered other outcomes related to cognition (cognitive impairment and cognitive decline) (30, 32). A 5-year longitudinal study revealed that physical impairment in individuals considered cognitively normal could lead to cognitive impairment clinically detectable only later and was associated with a greater risk of developing dementia of the AD type (46). However, it is important to emphasize that the causes of physical frailty and cognitive impairment are not well-established (47).

The etiology of frailty is possibly complex and might be multidimensional, including variables such as cognition, mood, nutrition, mobility, physical activity, strength, balance, endurance, coping, relationship, and social support, among other potential causes (47). Inflammation and oxidative stress are two factors that also play an important role in the development of both frailty and cognitive impairment (48). Frailty components have been linked to typical pathophysiological changes seen in AD (e.g., amyloid deposition) (13). However, it remains unclear whether the association is due to a direct (e.g., amyloid deposits are cause of frailty) or indirect (e.g., amyloid accumulation is related to frailty because they are both age-related conditions) mechanism.

At the same time, improved discrimination of neurodegenerative conditions from disturbances caused by disruption of the homeostatic balance (e.g., frailty; indirectly responsible for cognitive impairment) will impact clinical and research strategies (49). In particular, the impact of several operational definitions of frailty on cognitive decline has been attracting interest in this field of research. Cognitive frailty could be a heterogeneous clinical syndrome, characterized by concomitant physical frailty and MCI, while excluding cases with AD or other dementias (23). More recently, the construct of cognitive frailty proved capable of predicting short- and long-term all-cause mortality and overall dementia, particularly vascular dementia (31).

### Strengths and Limitations

These comprehensive meta-analysis results advance the literature beyond previously published integrative (50, 51) and/or systematic reviews (19–21) that have explored the relationship between cognitive impairment or dementia and frailty. The present review only included high-quality studies involving a prospective diagnosis of frailty according to validated criteria. Additionally, all studies reported the number of frail participants with incident cognitive disorders, while dementia diagnosis was based on established criteria.

Our data should be interpreted with caution because of potential limitations. First, the number of longitudinal prospective studies was limited. Second, most studies applied modified frailty criteria compared with the original. Third, significant heterogeneity was observed across the studies included in this review.

Lastly, in some studies, it is unclear whether the identification of participants with dementia resulted from a comprehensive assessment of cognitive and functional abilities (as required by current diagnostic criteria) or was merely based on global screening measures (e.g., the MMSE). Therefore, future research is required to understand how different operational definitions of frailty and cognitive impairment are useful and clearly defined as an integral concept.

Finally, frailty may represent a novel modifiable target in early cognitive impairment. Identification of modifiable risk factors for cognitive frailty will improve identification of high-risk individuals and help develop interventions to prevent cognitive decline in aging. Physical frailty and cognition together, in the absence of dementia, may have important implications in clinical settings and research scenarios worldwide.

## Author Contributions

IA: study design, meta-analysis, wrote, and reviewed the manuscript; MB and MarC: database management and search strategies, wrote, and reviewed the manuscript; MatC: study design and reviewed the manuscript.

### Conflict of Interest Statement

The authors declare that the research was conducted in the absence of any commercial or financial relationships that could be construed as a potential conflict of interest.
