# Supplementary material for: Frailty as a Predictor of Cognitive Disorders: A Systematic Review and Meta-Analysis
Source: Front Med (Lausanne). 2019 Feb 19;6:26. doi: 10.3389/fmed.2019.00026 (PMC6389599; doi:10.3389/fmed.2019.00026)
Supplement: Supplementary file 1 [file Data_Sheet_1.docx]

**Supplementary file. The complete list of excluded studies**

Aguilar-Navarro SG, Mimenza-Alvarado AJ, Anaya-Escamilla A, et al. Frailty and Vascular Cognitive Impairment: Mechanisms Behind the Link. Rev Invest Clin. 2016 Jan-Feb;68(1):25-32.

Alencar MA, Dias JM, Figueiredo LC, Dias RC. [Frailty and cognitive impairment among community-dwelling elderly.](http://www.ncbi.nlm.nih.gov/pubmed/23828529#_blank) Arq Neuropsiquiatr. 2013 Jun;71(6):362-7. doi: 10.1590/0004-282X20130039.

Apostolo J, Holland C, O'Connell MD, et al. Mild cognitive decline. A position statement of the Cognitive Decline Group of the European Innovation Partnership for Active and Healthy Ageing (EIPAHA). Maturitas. 2016 Jan;83:83-93. doi: 10.1016/j.maturitas.2015.10.008. Epub 2015 Oct 22.

Armstrong JJ, Godin J, Launer LJ, et al. Changes in Frailty Predict Changes in Cognition in Older Men: The Honolulu-Asia Aging Study. J Alzheimers Dis. 2016 Jun 15;53(3):1003-13. doi: 10.3233/JAD-151172.

Auyeung TW, Lee JS, Kwok T, Woo J. Physical frailty predicts future cognitive decline - a four-year prospective study in 2737 cognitively normal older adults. J Nutr Health Aging. 2011 Aug;15(8):690-4.

Avila-Funes JA, Amieva H, Barberger-Gateau P, et al. Cognitive impairment improves the predictive validity of the phenotype of frailty for adverse health outcomes: the three-city study. J Am Geriatr Soc. 2009 Mar;57(3):453-61. doi: 10.1111/j.1532-5415.2008.02136.x. Epub 2009 Feb 22

Bilotta C, Casè A, Nicolini P, et al. Social vulnerability, mental health and correlates of frailty in older outpatients living alone in the community in Italy. Aging Ment Health. 2010 Nov;14(8):1024-36. Doi: 10.1080/13607863.2010.508772.

Boyle PA, Buchman AS, Wilson RS, et al. Physical frailty is associated with incident mild cognitive impairment in community-based older persons. J Am Geriatr Soc. 2010 Feb;58(2):248-55. doi: 10.1111/j.1532-5415.2009.02671.x. Epub 2010 Jan 8.

Buchman AS, Boyle PA, Wilson RS, et al. Frailty is associated with incident Alzheimer's disease and cognitive decline in the elderly. Psychosom Med. 2007 Jun;69(5):483-9. Epub 2007 Jun 7.

Buchman AS, Schneider JA, Leurgans S, Bennett DA. Physical frailty in older persons is associated with Alzheimer disease pathology. Neurology. 2008 Aug 12;71(7):499-504. doi: 10.1212/01.wnl.0000324864.81179.6a.

Buchman AS, Bennett DA. Cognitive frailty. J Nutr Health Aging. 2013 Sep;17(9):738-9. doi: 10.1007/s12603-013-0397-9. Review

Buchman AS, Yu L, Wilson RS, et al. Brain pathology contributes to simultaneous change in physical frailty and cognition in old age. J Gerontol A Biol Sci Med Sci. 2014 Dec;69(12):1536-44. doi: 10.1093/gerona/glu117. Epub 2014 Aug 18.

Butler A, Gallagher D, Gillespie P. Frailty: a costly phenomenon in caring for elders with cognitive impairment. Int J Geriatr Psychiatry. 2016 Feb;31(2):161-8. doi: 10.1002/gps.4306. Epub 2015 Jul 1.

Cadore EL, Casas-Herrero A, Zambom-Ferraresi F, et al. Do frailty and cognitive impairment affect dual-task cost during walking in the oldest old institutionalized patients? Age (Dordr). 2015 Dec;37(6):124. doi: 10.1007/s11357-015-9862-1. Epub 2015 Dec 14.

Canevelli M, Cesari M. Cognitive frailty: what is still missing? J Nutr Health Aging. 2015 Mar;19(3):273-5. doi: 10.1007/s12603-015-0464-5. Review

Canevelli M, Cesari M, van Kan GA. Frailty and cognitive decline: how do they relate? Curr Opin Clin Nutr Metab Care. 2015 Jan;18(1):43-50. doi: 10.1097/MCO.0000000000000133. Review.

Cano C, Samper-Ternent R, Al Snih S, et al. Frailty and cognitive impairment as predictors of mortality in older Mexican Americans. J Nutr Health Aging. 2012 Feb;16(2):142-7.

Cesari M, Andrieu S, Rolland Y, et al. The cognitive impairment of frail older persons. J Nutr Health Aging. 2013 Sep;17(9):735-7. doi: 10.1007/s12603-013-0396-x.

Chen S, Honda T, Narazaki K, et al. Global cognitive performance and frailty in non-demented community-dwelling older adults: Findings from the Sasaguri Genkimon Study. Geriatr Gerontol Int. 2016 Jun;16(6):729-36. doi: 10.1111/ggi.12546. Epub 2015 Jun 16.

Chodosh J, Miller-Martinez D, Aneshensel CS. Depressive symptoms, chronic diseases, and physical disabilities as predictors of cognitive functioning trajectories in older Americans. J Am Geriatr Soc. 2010 Dec;58(12):2350-7. doi: 10.1111/j.1532-5415.2010.03171.x. Epub 2010 Nov 18.

Chong MS, Tay L, Chan M, et al. Prospective longitudinal study of frailty transitions in a community-dwelling cohort of older adults with cognitive impairment. BMC Geriatr. 2015 Dec 29;15:175. doi: 10.1186/s12877-015-0174-1.

Christensen K, Thinggaard M, Oksuzyan A, et al. Physical and cognitive functioning of people older than 90 years: a comparison of two Danish cohorts born 10 years apart. Lancet. 2013 Nov 2;382(9903):1507-13. doi: 10.1016/S0140-6736(13)60777-1. Epub 2013 Jul 11.

Dartigues JF, Amieva H. Cognitive frailty: rational and definition from an (I.a.N.a./i.a.g.g.) international consensus group. J Nutr Health Aging. 2014 Jan;18(1):95. doi: 10.1007/s12603-013-0437-5.

Doi T, Shimada H, Makizako H, et al. Mild Cognitive Impairment, Slow Gait, and Risk of Disability: A Prospective Study. J Am Med Dir Assoc. 2015 Dec;16(12):1082-6. doi: 10.1016/j.jamda.2015.07.007. Epub 2015 Aug 19

Espinoza SE, Jung I, Hazuda H. Frailty transitions in the San Antonio Longitudinal Study of Aging. J Am Geriatr Soc. 2012 Apr;60(4):652-60. doi: 10.1111/j.1532-5415.2011.03882.x. Epub 2012 Feb 8.

Feng L, Zin Nyunt MS, Gao Q, et al. Cognitive Frailty and Adverse Health Outcomes: Findings From the Singapore Longitudinal Ageing Studies (SLAS). J Am Med Dir Assoc. 2017 Mar 1;18(3):252-258. doi: 10.1016/j.jamda.2016.09.015. Epub 2016 Nov 9.

Gale CR, Ritchie SJ, Cooper C. Cognitive Ability in Late Life and Onset of Physical Frailty: The Lothian Birth Cohort 1936. J Am Geriatr Soc. 2017 Jun;65(6):1289-1295. doi: 10.1111/jgs.14787.

Halil M, Cemal Kizilarslanoglu M, Emin Kuyumcu M, et al. Cognitive aspects of frailty: mechanisms behind the link between frailty and cognitive impairment. J Nutr Health Aging. 2015 Mar;19(3):276-83. doi: 10.1007/s12603-014-0535-z. Review.

Han ES, Lee Y, Kim J. Association of cognitive impairment with frailty in community-dwelling older adults. Int Psychogeriatr. 2014 Jan;26(1):155-63. doi: 10.1017/S1041610213001841.

Hooghiemstra AM, Ramakers IHGB, Sistermans N, et al. Gait Speed and Grip Strength Reflect Cognitive Impairment and Are Modestly Related to Incident Cognitive Decline in Memory Clinic Patients With Subjective Cognitive Decline and Mild Cognitive Impairment: Findings From the 4C Study. J Gerontol A Biol Sci Med Sci. 2017 Jun 1;72(6):846-854. doi: 10.1093/gerona/glx003.

Kallenberg MH, Kleinveld HA, Dekker FW, et al. Functional and Cognitive Impairment, Frailty, and Adverse Health Outcomes in Older Patients Reaching ESRD-A Systematic Review. Clin J Am Soc Nephrol. 2016 Sep 7;11(9):1624-39. doi: 10.2215/CJN.13611215. Epub 2016 Jun 24. Review.

Kara O, Arik G, Sumer F, Ulger Z. Only Frailty? What About Other Factors Affecting Cognition? J Am Geriatr Soc. 2015 Jun;63(6):1281. doi: 10.1111/jgs.13461.

Kelaiditi E, Cesari M, Canevelli M, et al. Cognitive frailty: rational and definition from an (I.A.N.A./I.A.G.G.) international consensus group. J Nutr Health Aging 2013;17(9):726-34. doi: 10.1007/s12603-013-0367-2.

Kojima G, Taniguchi Y, Iliffe S, Walters K. Frailty as a Predictor of Alzheimer Disease, Vascular Dementia, and All Dementia Among Community-Dwelling Older People: A Systematic Review and Meta-Analysis. J Am Med Dir Assoc. 2016 Oct 1;17(10):881-8. doi: 10.1016/j.jamda.2016.05.013. Epub 2016 Jun 17. Review.

Kulmala J, Nykänen I, Mänty M, Hartikainen S. Association between frailty and dementia: a population-based study. Gerontology. 2014;60(1):16-21. doi: 10.1159/000353859. Epub 2013 Aug 17.

Landi F, Onder G, Cattel C, et al. Functional status and clinical correlates in cognitively impaired community-living older people. J Geriatr Psychiatry Neurol. 2001 Spring;14(1):21-7.

Lee JS, Auyeung TW, Leung J, et al. Physical frailty in older adults is associated with metabolic and atherosclerotic risk factors and cognitive impairment independent of muscle mass. J Nutr Health Aging. 2011 Dec;15(10):857-62.

Lee JS, Auyeung TW, Leung J, et al. Transitions in frailty states among community-living older adults and their associated factors. J Am Med Dir Assoc. 2014 Apr;15(4):281-6. doi: 10.1016/j.jamda.2013.12.002. Epub 2014 Feb 16.

Liao W, Hamel RE, Olde Rikkert MG, et al. A profile of The Clinical Course of Cognition and Comorbidity in Mild Cognitive Impairment and Dementia Study (The 4C study): two complementary longitudinal, clinical cohorts in the Netherlands. BMC Neurol. 2016 Nov 25;16(1):242.

Macuco CR, Batistoni SS, Lopes A, et al. Mini-Mental State Examination performance in frail, pre-frail, and non-frail community dwelling older adults in Ermelino Matarazzo, São Paulo, Brazil. Int Psychogeriatr. 2012 Nov;24(11):1725-31. doi: 10.1017/S1041610212000907. Epub 2012 Jun 1.

Malmstrom TK, Morley JE. Frailty and cognition: linking two common syndromes in older persons. J Nutr Health Aging. 2013 Sep;17(9):723-5. doi: 10.1007/s12603-013-0395-y.

Matthews FE, Arthur A, Barnes LE, et al. A two-decade comparison of prevalence of dementia in individuals aged 65 years and older from three geographical areas of England: results of the Cognitive Function and Ageing Study I and II. Lancet. 2013 Oct 26;382(9902):1405-12. doi: 10.1016/S0140-6736(13)61570-6. Epub 2013 Jul 17.

McGough EL, Cochrane BB, Pike KC, et al. Dimensions of physical frailty and cognitive function in older adults with amnestic mild cognitive impairment. Ann Phys Rehabil Med. 2013 Jul;56(5):329-41. doi: 10.1016/j.rehab.2013.02.005. Epub 2013 Mar 26.

Mhaoláin AM, Gallagher D, Crosby L, et al. Frailty and quality of life for people with Alzheimer's dementia and mild cognitive impairment. Am J Alzheimers Dis Other Demen. 2012 Feb;27(1):48-54. Doi: 10.1177/1533317511435661.

Mitnitski A, Fallah N, Rockwood K. A multistate model of cognitive dynamics in relation to frailty in older adults. Ann Epidemiol. 2011 Jul;21(7):507-16. doi: 10.1016/j.annepidem.2011.01.006.

Mitnitski A, Fallah N, Rockwood MR, Rockwood K. Transitions in cognitive status in relation to frailty in older adults: a comparison of three frailty measures. J Nutr Health Aging. 2011;15(10):863-7.

Monastero R, Palmer K, Qiu C, et al. Heterogeneity in risk factors for cognitive impairment, no dementia: population-based longitudinal study from the Kungsholmen Project. Am J Geriatr Psychiatry. 2007 Jan;15(1):60-9.

Mets T, De Deyn PP, Pals P, et al. COGNOS: care for people with cognitive dysfunction: a national observational study. Alzheimer Dis Assoc Disord. 2013 Apr-Jun;27(2):123-32. doi: 10.1097/WAD.0b013e318256d1d0.

Morley JE, Morris JC, Berg-Weger M, et al. Brain health: the importance of recognizing cognitive impairment: an IAGG consensus conference. J Am Med Dir Assoc. 2015 Sep 1;16(9):731-9. doi: 10.1016/j.jamda.2015.06.017.

Mulero J, Zafrilla P, Martinez-Cacha A. Oxidative stress, frailty and cognitive decline. J Nutr Health Aging. 2011 Nov;15(9):756-60. Review.

Naharci MI. Is weight loss an outcome of or a contributory factor for vascular dementia in frail elderly people? Med Princ Pract. 2015;24(3):298. doi: 10.1159/000377632. Epub 2015 Mar 5.

Ní Mhaoláin AM, Gallagher D, Crosby L, et al. Correlates of frailty in Alzheimer's disease and mild cognitive impairment. Age Ageing. 2011 Sep;40(5):630-3. doi: 10.1093/ageing/afr066.

Nikolova R, Demers L, Béland F. Trajectories of cognitive decline and functional status in the frail older adults. Arch Gerontol Geriatr. 2009 Jan-Feb;48(1):28-34. Epub 2007 Nov 5.

Panza F, Solfrizzi V, Frisardi S, et al. Different models of frailty in predementia and dementia syndromes. J Nutr Health Aging 2011;15(8):711-19.

Panza F, Solfrizzi V, Barulli MR, et al. Cognitive frailty - epidemiological and neurobiological evidence of an age related clinical condition: A systematic review. Rejuvenation Res. 2015;18(5):389-412. doi: 10.1089/rej.2014.1637. Epub 2015 Aug 20.

Panza F, Seripa D, Solfrizzi V, et al. Targeting cognitive frailty: Clinical and neurobiological roadmap for a single complex phenotype. JAD 2015;47(4):793-813. doi: 10.3233/JAD-150358. Review

Panza F, Lozupone M, Solfrizzi V, et al. Cognitive frailty: a potential target for secondary prevention of dementia. Expert Opin Drug Metab Toxicol. 2017 Oct;13(10):1023-1027. doi: 10.1080/17425255.2017.1372424. Epub 2017 Sep 5.

Raji MA, Al Snih S, Ostir GV, et al. Cognitive status and future risk of frailty in older Mexican Americans. J Gerontol A Biol Sci Med Sci. 2010 Nov;65(11):1228-34. doi: 10.1093/gerona/glq121. Epub 2010 Jul 9.

Robertson DA, Savva GM, Kenny RA. Frailty and cognitive impairment--a review of the evidence and causal mechanisms. Ageing Res Rev. 2013 Sep;12(4):840-51. doi: 10.1016/j.arr.2013.06.004. Epub 2013 Jul 4. Review.

Robertson DA, Savva GM, Coen RF, Kenny RA. Cognitive function in the prefrailty and frailty syndrome. J Am Geriatr Soc. 2014 Nov;62(11):2118-24. doi: 10.1111/jgs.13111. Epub 2014 Nov 4.

Rogers NT, Steptoe A, Cadar D. Frailty is an independent predictor of incident dementia: Evidence from the English Longitudinal Study of Ageing. Sci Rep. 2017 Nov 16;7(1):15746. doi:10.1038/s41598-017-16104-y.

Ruan Q, Yu Z, Chen M, et al. Cognitive frailty, a novel target for the prevention of elderly dependency. Ageing Res Rev. 2015 Mar;20:1-10. doi: 10.1016/j.arr.2014.12.004. Epub 2014 Dec 30. Review.

Ruan Q, D'Onofrio G, Sancarlo D, et al. Potential fluid biomarkers for pathological brain changes in Alzheimer's disease: Implication for the screening of cognitive frailty. Mol Med Rep. 2016 Oct;14(4):3184-98. doi: 10.3892/mmr.2016.5618. Epub 2016 Aug 9. Review.

Samper-Ternent R, Al Snih S, Raji MA, et al. Relationship between frailty and cognitive decline in older Mexican Americans. J Am Geriatr Soc. 2008 Oct;56(10):1845-52. doi: 10.1111/j.1532-5415.2008.01947.x. Epub 2008 Sep 22.

Sampson EL. Frailty and dementia: common but complex comorbidities. Aging Ment Health. 2012;16(3):269-72. doi: 10.1080/13607863.2012.657158. Epub 2012 Mar 2.

Sands LP, Yaffe K, Lui LY, et al. The effects of acute illness on ADL decline over 1 year in frail older adults with and without cognitive impairment. J Gerontol A Biol Sci Med Sci. 2002 Jul;57(7):M449-54.

Sargent L, Brown R. Assessing the Current State of Cognitive Frailty: Measurement Properties. J Nutr Health Aging. 2017;21(2):152-160. doi: 10.1007/s12603-016-0735-9. Review.

Scanlan JM, Binkin N, Michieletto F, et al. Cognitive impairment, chronic disease burden, and functional disability: a population study of older Italians. Am J Geriatr Psychiatry. 2007 Aug;15(8):716-24. Epub 2007 Jun 13.

Searle SD, Mitnitski A, Gahbauer EA, et al. A standard procedure for creating a frailty index. BMC Geriatr. 2008 Sep 30;8:24. doi: 10.1186/1471-2318-8-24.

Searle SD, Rockwood K. Frailty and the risk of cognitive impairment. Alzheimers Res Ther. 2015 Aug 3;7(1):54. doi: 10.1186/s13195-015-0140-3. eCollection 2015. Review.

Shatenstein B. Frailty and cognitive decline: links, mechanisms and future directions. J Nutr Health Aging. 2011 Aug;15(8):665-6.

Shimada H, Makizako H, Doi T, et al. Combined prevalence of frailty and mild cognitive impairment in a population of elderly Japanese people. J Am Med Dir Assoc. 2013 Jul;14(7):518-24. doi: 10.1016/j.jamda.2013.03.010. Epub 2013 May 10.

Solfrizzi V, Scafato E, Frisardi V, et al. Frailty syndrome and all-cause mortality in demented patients: the Italian Longitudinal Study on Aging. Age 2012 Apr;34(2):507-17. doi: 10.1007/s11357-011-9247-z. Epub 2011 Apr 26.

Takechi H, Sugihara Y, Kokuryu A, et al. Both conventional indices of cognitive function and frailty predict levels of care required in a long-term care insurance program for memory clinic patients in Japan. Geriatr Gerontol Int. 2012 Oct;12(4):630-6. doi: 10.1111/j.1447-0594.2011.00828.x.

Wang C, Ji X, Wu X, et al. Frailty in Relation to the Risk of Alzheimer's Disease, Dementia, and Death in Older Chinese Adults: A Seven-Year Prospective Study. J Nutr Health Aging. 2017;21(6):648-654. doi: 10.1007/s12603-016-0798-7

Wilkins CH, Roe CM, Morris JC, Galvin JE. Mild physical impairment predicts future diagnosis of dementia of the Alzheimer's type. J Am Geriatr Soc. 2013 Jul;61(7):1055-9. doi: 10.1111/jgs.12255. Epub 2013 May 6

Woods AJ, Cohen RA, Pahor M. Cognitive frailty: frontiers and challenges. J Nutr Health Aging. 2013 Sep;17(9):741-3. doi: 10.1007/s12603-013-0398-8.

Wu YH, Liu LK, Chen WT, et al. Cognitive Function in Individuals With Physical Frailty but Without Dementia or Cognitive Complaints: Results From the I-Lan Longitudinal Aging Study. J Am Med Dir Assoc. 2015 Oct 1;16(10):899.e9-16. doi: 10.1016/j.jamda.2015.07.013. Epub 2015 Aug 28.

Yassuda MS, Lopes A, Cachioni M, et al. Frailty criteria and cognitive performance are related: data from the FIBRA study in Ermelino Matarazzo, São Paulo, Brazil. J Nutr Health Aging. 2012 Jan;16(1):55-61.
